# Going green: Impact of green supply chain management practices on sustainability performance

**DOI:** 10.3389/fpsyg.2022.973676

**Published:** 2022-11-15

**Authors:** Azhar Ahmad, Amir Ikram, Muhammad Farooq Rehan, Ayyaz Ahmad

**Affiliations:** ^1^Institute of Business and Management, University of Engineering and Technology, Lahore, Pakistan; ^2^Lyallpur Business School, Government College University Faisalabad, Faisalabad, Pakistan; ^3^Institute of Quality and Technology Management, University of the Punjab, Lahore, Pakistan

**Keywords:** green supply chain management practices, sustainability performance, institutional pressure, green manufacturing, green information system

## Abstract

The rising environmental challenges are capturing the attention of stakeholders of corporations due to increasing competition. The sustainable performance of firms does not merely gauge their economic performance but also their sustainable development. Since the environmental supply chain management of firms has an important part in maneuvering their sustainable performance, the present study focuses on assessing the impact of green supply chain management practices on the sustainable performance of the textile, automobile, and tobacco industries. The data were collected from 384 organizations and analyzed through SPSS and AMOS. The results reveal that the impact of green manufacturing, green purchases, eco-design, and green information system are significant and positive on the sustainable performance of the organizations, whereas the impact of cooperation with customers is insignificant. Similarly, the moderation of institutional pressures between green manufacturing, green purchases, eco-design, and sustainability performance is insignificant, whereas it significantly moderates between cooperation with customers, green information systems, and sustainable performance. The study is significant and novel for considering five different factors of green supply chain management (GSCM): green manufacturing, green purchasing, cooperation with customers, eco-design, and green information systems. Furthermore, adding institutional pressures as a moderator between GSCM and sustainable performance adds to the novelty and uniqueness of the research. The managerial implications have also been discussed.

## Introduction

With an increasing concern for the environment as a major determinant of a company’s sustainability, it is imperative to investigate going green behaviors in the context of emerging economies. By examining both economic and non-economic aspects, a firm’s long-term performance is referred to as its values, communication, and strategies (Schaltegger and Wagner, 2017). Firms’ execution of corporate social responsibility is also reflected in their long-term performance. Furthermore, the firms’ long-term viability is assessed using three main components: economic performance indicators; social performance indicators; and environmental performance indicators (Muhammad et al., 2020). The sustainable performance of firms does not merely gauge their economic performance but overall sustainable development. The rising environmental challenges are capturing the attention of stakeholders of corporations due to increasing competition. Currently, businesses are concerned about the environmental consequences of their products to enhance their sustainable development. Green supply chain management practice in the manufacturing industry also contributes to enhancing the long-term values of the company (Ananda et al., 2018). The industrial sector, particularly the manufacturing sector, is the main contributor to CO_2_ and greenhouse gas emissions. Therefore, environmental management and green innovation are vital for the industry to deliver its corporate social responsibility. Moreover, the importance of GSCM in the sustainable performance of a firm is unambiguously supported by many studies.

Supply chain management practices are deeply linked to the satisfaction of the customers. For that purpose, it needs coordination and integration of the business framework. The business process, which must be cohesive, includes manufacturing, purchasing, marketing logistics, and information system. Therefore, the supply chain management practices focus on the customers’ responses, quality, and environmental sustainability (De et al., 2020). The identification and choice of practices are necessary for having a competitive advantage at the end of supply chain management (Ikram et al., 2018). Literature is filled with studies conducted on multinational organizations and large enterprises that examine the impact of green practices, such as green production and green purchasing, green logistics, green design, and green distribution on sustainable performance (Ananda et al., 2018). Besides research domains, manufacturing organizations have started implementing environment-friendly management practices called green supply chain management (GSCM) practices. These practices are expected to reduce air emissions, solid wastes, effluent waste, and other toxic tangible or intangible materials in manufacturing industries. In addition, to improving performance sustainability, these practices have a strong impact on profitability and market share (Tiwari et al., 2018).

In the last few years, a rapid interest in green manufacturing has been observed in both the practical and research fields of Pakistani manufacturing firms. On the operational level, green manufacturing practice focuses on the workers and machines, while on the process level, it keeps a check on control and planning, whereas the system-level practices involve implementation strategies and designs (Govindan et al., 2015). According to current surveys and studies, it has been noted that business alliances and buyers are abandoning the manufacturers whose goods and services are not eco-friendly (Gupta, 2019). This practice is growing quite popular among the manufacturing business and the customers. Apart from the advantages in market share and customer consumption, green manufacturing is directly linked with the sustainable performance of the organizations.

Green purchasing is another important practice under the cover of green supply chain management. In recent years, the notion of purchasing has been thought of as a bottom-line financial consideration, but recent research on purchasing practices indicates that purchasing is also deeply linked with environmental management. Globally it has been noted that the eco-friendly criteria of purchasing have a significant impact on the improved performance of organizations along with economic and environmental concerns (Liobikienė et al., 2016). There are numerous green purchasing strategies that firms adopt. The strategies differ in their nature and have different impacts on the environmental behaviors of providers. It focuses on products that have more eco-friendly attributes. This strategy is highly valuable as the consumers are more likely to prefer environment-friendly products and consequentially benefit the organization in the form of increased market share (Khoiruman and Haryanto, 2017).

Regarding eco-friendly products, eco-design has emerged as a significant strategy in the business management domain of Pakistan. The concept of eco-design was established by World Business Council for Sustainable Development, and since that time, it has been influencing the whole cycle of a product (Fernando, 2017). The production, packaging, and distribution of products are designed according to eco-design strategies. Among all the advantages, the sustainable performance of the organization is noteworthy. As far as the sustainability performance of the firm is concerned, eco-design gives a better environmental commitment and, therefore, a momentous practice in green supply chain management.

The involvement of technology in manufacturing businesses often comes up with environmental hazards. To eliminate environmental issues, green information systems emerged as a significant practice in green supply chain management. The environment sustainability regarding the green information system includes the processes, software, and other related technologies to facilitate the sustainable performance of a firm, and support its individual, organizational, social, and environmental goals (Recker, 2016). Information plays an important role in green information systems. Green information helps in product cycle analysis, environmental management, and the design of eco-friendly practices. The effectiveness of green information systems hugely depends on the IT capacities of the firm (Anthony, 2016). Despite its importance in management practices, the existing research on this area is narrow and requires deep investigations so that organizations can benefit themselves with a green information system. The rising importance of GSCM practice has shifted the attention of policymakers from economic performance to sustainability performance. The reason is that later consider the wider prospects of the company’s development such as development in environmental protection. Non-compliance with environmental laws and regulations in the company negatively impacts sustainability performance. Moreover, modern statistical tests are conducted to analyze these factors that have no traces in the existing research bodies regarding the Pakistani context. Due to the limited studies available in the manufacturing context of Pakistan, the present study acknowledged the need to study these supply chain management practices in relation to sustainability performance. The role of the institution also seemed neglected in past studies; therefore, the moderating role of institutional pressure is examined in association with the GSCM practices and sustainability performance.

The study intends to cover the gap in the literature by empirically investigating the impression of the components of GSCM practices on the sustainability performance of the manufacturing industry in Pakistan through the moderating role of institutional pressure. The study also feeds back to the literature on sustainability performance by debating how the green supply chain facilitates firms to achieve long-run sustainability performance growth. The novelty of this study is to use primary data by conducting surveys from the manufacturing sectors of Pakistan, including the textile, sugar, automobile, and tobacco industries, to explain sustainability performance through GSCM and its components. Few studies in the literature have empirically examined the impact of GSCM on, sustainability performance but research on the moderating role of institutional pressures between GSCM and sustainability performance is limited. This study objects to filling lacunas in the literature by conducting primary data on manufacturing industry sustainability performance.

The present study examines the impact of components of green supply chain management on the sustainability performance of the manufacturing industry of Pakistan. The gaps in the experiential research of sustainability, performance are filled by executing the following objectives: (1) examine the impact of green manufacturing and green purchasing on the sustainability performance, (2) examine the role of cooperation of customers in the sustainability performance, (3) examine the role of eco-design and green information systems on sustainability performance, and (4) analyze the moderating role of institutional pressure on the relationship between GSCM practices and sustainability performance.

This study focuses on all the five components of green supply chain management such as green manufacturing, green purchases, cooperation with customers, eco-design, and green information systems to explain the sustainability performance of the manufacturing industry of Pakistan. This research will play a key role in enunciating the policymakers about the constructive role of the green supply chain in enhancing the sustainability performance of the manufacturing industry of Pakistan. The discouraging performance of the manufacturing industry in Pakistan is gauged on the scale of the economic downturn, whereas environmental consequences of the industry also have repercussions on sustainability performance. Provided this backdrop, this research also aims to provide an exclusive perception of how to inform policymakers and environmentalists about the role of the green supply chain in the industry’s sustainable performance. The rest of the article is structured as follows. The research article first reviews the existing literature. The second part outlines the methodology employed in the study for testing the conceptual model, and the third part of the study describes the analysis and findings. Discussion and conclusion are the end part of the research article.

### Literature review

The supply chain management concept states the management of inventory, the relationship between suppliers and buyers, procurement, production, and activities associated with the production of goods (Rao and Holt, 2005; Ikram et al., 2018). Similarly, green supply chain management refers to managing all activities in the firm by considering the environmental consequences of those activities to assure environmentally friendly production and delivery processes. Various studies in literature support the positive role of GSCM in the sustainability performance of the industry (Awan et al., 2017; Khan and Qianli, 2017; Khan et al., 2019). Corporations are inclined to adapt the GSCM to enhance their competitiveness and sustainability performance (Rao and Holt, 2005; Yang et al., 2013).

With the growing concern for the environment, reducing costs and improving quality products have become the target for various businesses. There is now a focus on green production from product development up to the management of every step of the product life cycle. The environmental practice steps may involve eco-designs, recycling processes, reuse of the products with minimal cost expenditure, and clean production (Chavez et al., 2016). The literature on environmental management suggests that green operations are related to both products and their related practices of the environment. This reduces product damage and positively affects the supply chain processes involving natural resources (Choi and Hwang, 2015). The capacity of an organization to carry out its environmental practices and sustainable operations is highly important for its performance output. The value of green manufacturing and production has been well recognized in the literature, but the focus on the upstream suppliers is not enough for their performance enhancement role. This supply chain management system helps reduce ecological hazards and the burden on the environment from product production and disposal. This also aids in gaining profits for the manufacturing units and gives them a competitive edge (Arshad Ali et al., 2020).

In the world of today, the focus is being shifted toward organic foods, green technology, green products, and sustainable energy. Literature highlights the reasons that can be behind the purchase of these green products as why consumers buy green products or are involved in making more eco-friendly choices (Arshad Ali et al., 2020). These choices provide a win-win situation for the planet and its living beings. It has also been observed that consumers are willing to buy expensive but eco-friendly products, which gives a big opportunity to the government to make policy changes that are more eco-friendly (Peattie, 2001). An empirical investigation of Chinese consumers suggested that value-attitude-behavior are the three basic constructs behind any purchase behavior (Keles and Bekimbetova, 2013).

Eco-design can be defined in terms of both principle and approach. It prioritizes the criteria of the protection of the environment before the life cycle of the product or service. The basic logic behind eco-designs is the minimization of environmental hazards and incorporation of eco-design in the manufacturing, usage, and disposal of products. The successive stages of a product life cycle in which eco-design can be incorporated include raw material extraction and supply, then its manufacturing, proceeding product distribution, and finally customer use. The eco-design approach adopted by the organizations starts from raw material extraction and goes up to retailers and consumers. The processes involving the product’s value chain are also included in the eco-design (Arshad Ali et al., 2020). Environmental sustainability includes the human attitude and the social, organizational, and environmental perspectives, so we can say that both micro and macro issues are involved in it, as stated by Melville. There has begun research in the newly developed area of green IT but more is still to be learned (Jenkin et al., 2011). The reason behind the organization choosing green IT for their systems is the reduction of costs during operating procedures and the minimization of environmental hazards caused during various business procedures (Dedrick, 2010). Moreover, the bigger reasons behind the adoption of green IT are the essential benefits like employee self-esteem and good corporate citizenship. This also provides a better image of the corporate and the regulation pressure. The success of green IT services needs further research to support the arguments regarding the involvement of employees in the buy-in and the environmental initiatives (Jenkin et al., 2011).

Literature mentions the institutional theory, which is helpful in the investigation of how the forces of an institution lead an organization to be receptive to the needs of others in society. Institutional pressure occurs in three different ways named as normative, mimetic, or coercive. A study on the investigation of the influence of mimetic and coercive pressures on managers’ behavior was checked after the adoption of the green information system. The organization’s response toward uncertainty when the path of action is not clear is termed mimetic isomorphism. This usually occurs when another organization adopts a policy or a technology and gets positive influence from it (Kaur, 2021). Whereas coercive isomorphism responds to both formal and informal pressures from other organizations, such as those in supply chain management. Thus, we can say that the behavior of the senior managers was influenced by the adoption of the green information system because it caused pressure from higher authorities, supply chain suppliers, and consumers as well. In the regulated field, the most often seen pressure is coercive pressure. Therefore, the issues on the environment are taken as negative externalities as they force the managers appointed at senior levels to enhance the environmental performance of the organization. All the firms face coercive pressure at the same level that leads them toward regulating better adaptive procedures. Thus, the managers that adopt green IS through coercive pressure will develop positive behaviors toward it as both environmental and commercial advantages are obtained *via* the adoption of green IS (Ananda et al., 2018).

### Resource base and institutional theory

We empirically investigate the impacts of green supply chain management methods on sustainability performance, as well as the moderating effects of institutional pressure, while drowning in institutional theory and the resource-based perspective. Resource base theory is a key concept in strategic management that has gained popularity in complementary fields like supply chain management and management sub-disciplines like entrepreneurship (Hitt et al., 2016; Kuenzi et al., 2019). Barney (2012) suggested that according to resource-based theory, supply chain management will frequently possess characteristics that enable it to be a source of long-term competitive advantage. The issue regarding the supply chain resources being upstream and internal resource development can be taken under enhanced scrutiny. Some researchers have confirmed that the attributes of resource base theory align inside the supply chain management and act as a weapon of competition. Currently, there have been struggles to categorically realize the link between RBT and consistent supply chain management (Peattie, 2001). With the help of sustainability, the firms can create and develop their market image and reputation, which helps them to improve their marketability and gain immense profits from their products and services. This is essential for the organization as it enables them to gain a competitive edge against other suppliers rather than a stand-alone firm. RBT suggests that the demands and resources from which the products are made are highly valuable and hard to copy and have no alternative, thus, giving the firm’s success in the long run (Hitt et al., 2016).

Hirsch (2008) suggested that the impact of the institutional environment on organizational behavior can be investigated using institutional theory. According to Oliver (1997), the logic of the resource-based view has not inspected the social context in which resource selection decisions are entrenched. Thus, Oliver (1997) suggests a theoretical framework built on the merger of the resource-based perspective and institutional theory to solve the shortcomings of the resource-based approach. Institutional theory has been heavily utilized in the development of frameworks for green supply chains as well as the adoption of high-quality initiatives and technological applications (Sarkis et al., 2011). When the incentive for the adoption of behaviors or technologies derives from legitimacy, the institutional theory provides a more comprehensive explanation (DiMaggio and Powell, 1983). We argue that institutional theory, out of all organizational theories, should be the second theory chosen because it provides the best justification for the social and environmental components of supply chain sustainability performance (Seles et al., 2016).

### Impact of green manufacturing on sustainability performance

Green supply chain management has drawn the attention of academics and business professionals due to growing environmental concerns. Green manufacturing has some standards that need to be ensured during its manufacturing like no significant safety problems, no health coercions on the workers and product operators, and no environmental pollution, waste recycling, and waste disposal (Gong et al., 2019). Green manufacturing in the industry comprises the planning of manufacturing and management to check the usage of energy, greenhouse gas emissions, likewise waste defecation. Green manufacturing works to reduce the municipal discarded by growing the efficiency of firms (Aziziankohan et al., 2017). Considering the viewpoints of institutional theory and resource base theory, the following hypotheses are put out to investigate how different institutional pressures lead to firms over time integrating similar risk management practices into their supply management processes.

**H1:** Green manufacturing has a significant impact on the sustainability performance of the manufacturing industry in Pakistan.

### Impact of green purchases on sustainability performance

Green purchases show the efforts put into the economic growth of a country and its development while ensuring that the sources utilized in their manufacture are still there and the environmental services. The demand for a green economy is the operational policy agenda that aids in the progress measure at the edge of both economies and the environment (Cherian and Jacob, 2012). It can help in economic diversification by speeding up the rate of technological changes and sustainability performance. Green purchases practice refers to the firms’ procurement management to control the waste defecation in form. Green purchases align with the sustainability performance of firms by winning a repute in the market (Liobikienė et al., 2016; Khoiruman and Haryanto, 2017). Moreover, green purchases facilitate the long terms sustainability performance of firms by assuring that the purchased products of firms are environment friendly (Cherian and Jacob, 2012). The following hypothesis is built in light of the aforementioned studies:

**H2:** Green purchasing has a significant impact on the sustainability performance of the manufacturing industry in Pakistan.

### Impact of cooperation with customers on sustainability performance

Customers are the main stakeholder of the company, which can influence the firm’s practices and production procedures (Chavez et al., 2016). Customers being key economic agents, can exert pressure on firms to circumvent environmental degradation by opting for environment-friendly practices (Dhull and Narwal, 2016). Strong cooperation with customers enhances the GSCM in organizations which consequently increases the efficiency and sustainability performance of firms. It is important for management to indulge all the stakeholders, particularly customers, to promote GSCM practice in a company (Neramballi et al., 2017). The association between supplier evaluation and supplier selection is insignificant to sustainability performance. The factor of cooperation with customers was also considered and it was found to have a significantly negative association with sustainability performance (Foo et al., 2018). According to the resource-based theory, when demand increases, performance increases, and customers are the main agents behind the demand and supply balance increase. We can say that in relevance to the RBT, cooperation with customers will significantly impact sustainability performance. Thus, based on the above literature following hypothesis is recommended:

**H3:** Cooperation with customers significantly impacts the sustainability performance of the manufacturing industry in Pakistan.

### Impact of eco-design on sustainability performance

Eco-design is defined as the production process designs of firms to check the environmental degradation and negative impact on the ecosystem (Kuo et al., 2018). Eco-design practices to control the environmental hazards of the production process will increase the efficiency and productivity of firms. These actions include efficient usage of friendly energy sources in the manufacturing process, which intensifies the productivity in the organizations (Aziziankohan et al., 2017). The applications of green product and procedure design include using eco-friendly raw materials and eco-design that has decreased energy and material consumption. Thus, based on the above literature following hypothesis is proposed:

**H4:** Eco-design has defined a significant impact on the sustainability performance of the manufacturing industry in Pakistan.

### Impact of green information systems on sustainability performance

The green information system deals with the employment of the information system to support environment-friendly operations and sustainable performance (Recker, 2016; Shevchuk and Oinas-Kukkonen, 2016). The system optimizes the firms’ behaviors and activities toward clean energy and green innovations. The green information system is a key component of GSCM which coordinates green supply chain processes (Seethamraju and Frost, 2019). The practice of green information systems increases the modest advantage and performance of firms, which positively influences sustainability performance (Chen, 2008; Dao et al., 2011). This is because it minimizes energy consumption as compared to traditional IT systems. Green IT is far more important than just reducing energy consumption (Dao et al., 2011). The resources will be shifted toward the natural ones and more focus will be given to environment-friendly manufacturing practices. Hence, on the basis of the above literature following hypothesis is recommended:

**H5:** Green information System has a significant impact on the relationship between the performance of the manufacturing industry.

### Moderating impact of institutional pressure between green manufacturing and sustainability performance

Institutional pressures on organizations to comply with the rules and regulations to protect the environment further reinforce the connection between green manufacturing and sustainability performance (Sarkis et al., 2011). Seethamraju and Frost (2019) indicated that in their study that by using data from 396 manufacturers in China to examine the link between institutional pressure and GSCM. All types of institutional pressure cause no harm yet contribute toward a win-win situation for all the organizations (Sarkis et al., 2011). According to the resource-based theory, when the demand in a green supply chain increase, the resources need to be increased for better sustainability. When there is institutional pressure, organizations tend to deliver better performance in green manufacturing and green production, etc. Hence, the following hypothesis is proposed:

**H6:** Institutional pressure has a moderating impact between green manufacturing and sustainability performance of the manufacturing Industry in Pakistan.

### The moderating impact of institutional pressure on green purchases, and sustainability performance

The institutional quality in the country will be able to exert institutional pressures on firms to adopt green purchases by recycling waste and controlling waste defecation (Habib et al., 2021). The institution pressure will render the firm to show long-term sustainability performance by dealing with the environmental impacts of firms. Abid (2017) also proposed in his study that institutional quality and financial development highly matter for environmental quality and greenhouse gas emission. Green purchasing capabilities have a significant impact on GSCM practices. According to the resource-based theory, green purchases will increase the demand for green manufacturing, and thus, the firms will get a competitive edge (Foo et al., 2018). The institutional pressure can significantly impact the green purchase, as discussed above, thus, the following hypothesis is suggested:

**H7:** Institutional pressure has a moderating impact on the relationship between green purchasing and the sustainability performance of the manufacturing industry in Pakistan.

### Moderating the impact of institutional pressure on cooperation with customers and sustainability performance

Institutional pressure safeguards the rights of customers by assuring the compliance of the company’s rights toward key stakeholders (Kaur, 2021). The company practicing the GSCM will tend to cooperate with customers to improve the company’s reputation. Moreover, good institutional quality in the country will also promote the customers’ rights by keeping an eye on the company’s conduct. The study also found that customer pressure act as a moderator in its impact on the institutional factors of cooperative practices, but it has no significant relationship with coercive practice (David and Muthini, 2019). According to the resource-based theory, when the resources are enhanced, supply chain management practices are strengthened. Customer cooperation can significantly influence the sustainability performance of the firms, but the cooperation by the customer is affected by various institutional pressures, so the role of institutional pressure in this relationship can be highly significant. Therefore, in the light of above discussion following hypothesis is built:

**H8:** Institutional pressure has a moderating impact on the relationship between cooperation with customers and sustainability performance of the manufacturing industry in Pakistan.

### Moderating the impact of institutional pressure on eco-design and sustainability performance

Institutional pressure also stresses the environment-friendly procedures of producing goods and services in the company (Dubey et al., 2015). Institutional pressures on firms to circumvent inflicting hazards to the environment will also facilitate the firm to enhance its efficiency and competitiveness (Khan and Qianli, 2017). It was concluded that institutional pressure has a substantial positive effect on supply chain relationship management and sustainable supply chain design and coercive pressure, normative pressure, and mimetic pressure have various levels of negative moderating impacts (Habib et al., 2021). Institutional pressure has a varying degree of influence on supply chain management practices, as proved by the above-mentioned studies. Thus, the following hypothesis is proposed in light of the research mentioned above:

**H9:** Institutional pressure has a moderating impact on the relationship between eco-design and the sustainability performance of the manufacturing industry in Pakistan.

### The moderating impact of institutional pressure on the green information system, and sustainability performance

**FIGURE 1:**
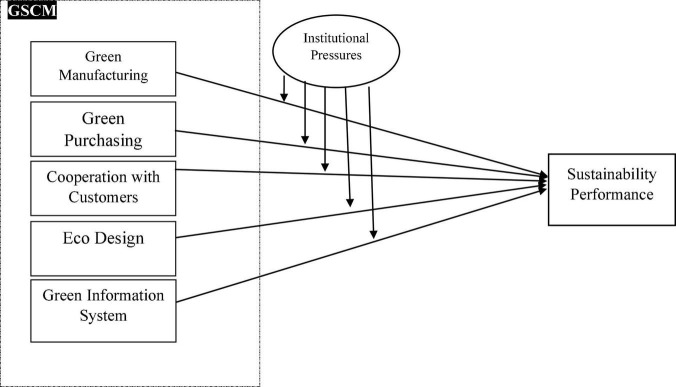
Theoretical framework.

Institutional pressure on the company will render company to use green information systems to promote environment-friendly operations. Environment protection programs are launched in various countries, which local institutions successfully execute. Internal environmental management organizations reinforce the practice of GSCM in various industries (Khan et al., 2019). According to the resource-based theory, when the resources are valuable and irreplaceable, it gives the firm a competitive advantage. The implementation of green IS in the green supply chain management practice will help in the development of sustainability performance, but various levels of institutional pressure will influence it and this impact can be highly significant (David and Muthini, 2019). Based on the results of the literature, the study suggests the following hypothesis:

**H10:** Institutional pressure has a moderating impact on the relationship between the green information system and the sustainability performance of the manufacturing industry in Pakistan. In light of the above discussion, the theoretical framework of the study has been depicted in Figure 1.

## Research methodology

Population and sampling are the major concerns to be looked at by the researcher while collecting the data for the research process. The population is the targeted respondents from whom the researcher would collect the data. The population is the observed set of respondents from which the researcher had to select the sample for studying (Van Blerkom, 2008). As the population in this study is unknown, it is recommended by Krejcie and Morgan (1970), the sample size for this type of population be 384. In the present study, a survey strategy was adopted to collect the data from the respondents through a structured questionnaire. The questionnaire used was adopted from the previous studies, and it was used after being examined by experienced professionals in the research field.

## Data analysis and results

To empirically explore the relationship of GSCM with sustainable performance, the study used descriptive analysis, confirmatory factor analysis (CFA), and structural equation modeling (SEM). Descriptive analysis was performed by using the software SPSS in which descriptive statistics are calculated for variables, including mean, median, mode, and standard deviation. However, to test the structural relationship among the variable, the SEM technique is performed by using the software of AMOS. The CFA is a multivariate empirical analysis technique used in this study to measure the relationship of the variables with latent variables in which convergent and discriminant validity is measured. Moreover, regression analysis is also done.

### Confirmatory factor analysis

Table 1 shows the results of the confirmatory factor analysis, and the values for the goodness of fit index, CMIN value, incremental fit index, competitive fit index, and root mean square error of approximation are presented below:

**TABLE 1:** Model fitness indices.

CFA indicators	CMIN/DF	GFI	IFI	CFI	RMSEA
Threshold value	≤3	≥0.80	≥0.90	≥0.90	≤0.08
Observed value	1.788	0.849	0.957	0.956	0.049

**FIGURE 2:**
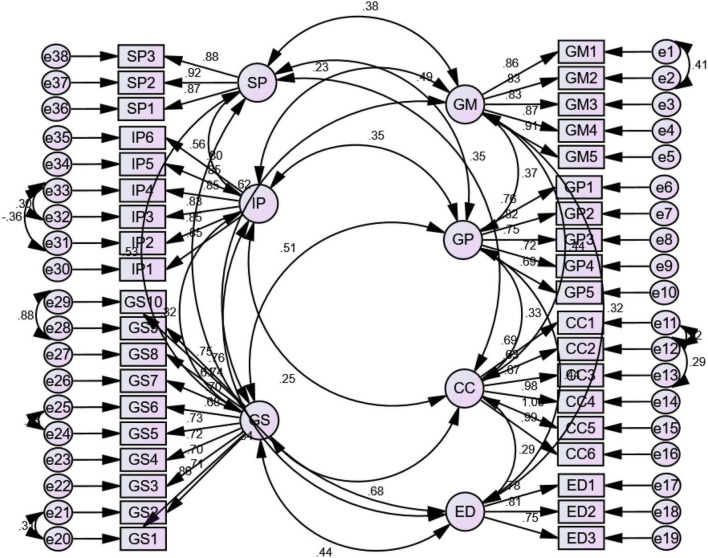
Confirmatory factor analysis.

The threshold value for CMIN to be less than or equal to 3; In this case, it can be observed that the observed value is 1.788, which is exactly according to the threshold value, in the case of the value of the goodness of fit index, the value should be more than or equal to 0.80, and in this case, the value is 0.849, which is exactly according to the threshold value. Whereas the value for root mean square error of approximation should be less than 0.08 or equal to 0.08, and in this case, it is equal to 0.049, which depicts the reliability, convergent, and discriminant validities. Figure 2 represents the screenshot of the confirmatory factor analysis.

### Structural equation modeling

Table 2 represents the results of structural equation modeling; a multivariate statistical analysis method was applied to check for the structural relationships present between the variables.

**TABLE 2:** Structural equation modeling.

			Estimate	S.E.	C.R.	*P*
**Regression**						
SustainPerf	<—	GreenManu	0.137	0.050	2.634	0.008
SustainPerf	<—	GreenPurc	0.163	0.056	3.366	0.000
SustainPerf	<—	CoopCust	–0.010	0.065	–0.168	0.866
SustainPerf	<—	EcoDesign	0.325	0.054	6.971	0.000
SustainPerf	<—	GreenInfSys	0.250	0.081	3.617	0.000
**Moderation**						
ZSustainPerf	<—	IPxGM	–0.047	0.040	–1.005	0.315
ZSustainPerf	<—	IPxGP	0.040	0.039	0.856	0.392
ZSustainPerf	<—	IPxCC	0.101	0.038	2.183	0.029
ZSustainPerf	<—	IPxED	–0.003	0.041	–0.056	0.955
ZSustainPerf	<—	IPxGS	0.151	0.011	3.265	0.001

**FIGURE 3:**
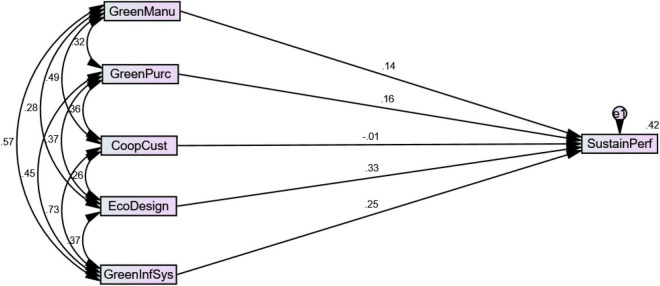
Structural equation modeling.

Figure 3 shows the path diagram of the structural equation modeling that represents the direct impacts of the variables and the indirect impacts of the variables on each other.

## Conclusion and implications of the study

The research aims to know about the impact of green manufacturing, green purchases, cooperation with customers, and green information systems on sustainable performance. The study aims to study sustainable performance with respect to the organization and the operating activities of the business as well. Moreover, the current study has taken out the moderating concept of institutional pressure. The study has taken sustainable performance as a dependent variable. For the sake of checking the results, the study has proposed some hypotheses as well. First, the study has proposed that the “Impact of green manufacturing on sustainability performance is significant.” This hypothesis was accepted by the researchers. The results of the study have highlighted that with an increase in green manufacturing the sustainability performance of organizations can be enhanced significantly. Abdul-Rashid et al. (2017) argued that production wastage and improper manufacturing are causing different problems for society. That is why the researcher has appreciated incorporating green manufacturing in the production processes to attain sustainable performance throughout the business processes.

Furthermore, different studies have quoted that sustainability performance can also result in economically sound and beneficial for organizations as well. However, another study by Cankaya and Sezen (2018) suggested that green manufacturing can play a role significantly for future growth. The practices of green manufacturing can also establish future certainty and will also help in cutting the cost and the wastage of production will be lower as well. This relationship has been supported by prior studies as well. The study of Chin et al. (2015) suggested that in the growth of an economy, green purchase plays a significant role, as it has got significant importance in the recent decade with respect to scholarly studies.

Moreover, De et al. (2020) explained that in the economic development of any economy green purchases also played a moderating role as it involves futuristic practices which can be adopted by any organization that is looking for sustainability that is why the researcher has considered this relationship as significant and positive. The third hypothesis proposed by the study was that the “impact of cooperation with customers on sustainability performance is significant.” This hypothesis was rejected by the study because of the insignificant value shown by the analysis of this hypothesis. This statement was accepted by the results that have shown the positive value of the relationship. The study has taken the supporting literature on this relationship, explaining that eco-design can be recognized as a design for environmental safety and production publicity concurrently. Furthermore, eco-design can be considered a design for environmental or green design of the organizational processes to minimize the effects of business processes on the environment and society.

Pigosso et al. (2014) found that incorporating eco-design into each business operation can improve sustainability performance. The researcher investigated the impact of the green information system on sustainability and offered a hypothesis that was accepted by the study. The study suggests that the moderating influence of institutional pressure on green manufacturing and sustainability performance is important. Rodrigues et al. (2016) found institutional pressures on businesses to follow rules and regulations to preserve the environment from additional harm, as well as a link between green manufacturing and sustainability performance. The study of Schöggl et al. (2017) highlighted institutional pressure on firms to adopt green purchases by recycling and wasting waste defecation. Moreover, they highlighted that the institutional pressure would render the firms to show that this pressure cannot lead to higher sustainability performance in the long run. Zaid et al. (2018) proposed that good institutional pressure on quality will also promote the factor of cooperation with customers and this will cause better sustainability performance with respect to institutional stake in the organizations. Future researchers can examine the theory of planned behavior in the context of green purchase behavior. Moreover, it might be interesting to examine the potential role of sustainability and digital transition issues with respect to going green, as suggested in the recent literature (Centobelli et al., 2020, 2022; Cerchione and Bansal, 2020).

## Data availability statement

The raw data supporting the conclusions of this article will be made available by the authors, without undue reservation.

## Ethics statement

Ethical review and approval was not required for the study on human participants in accordance with the local legislation and institutional requirements. Written informed consent from the patients/participants or patients/participants legal guardian/next of kin was not required to participate in this study in accordance with the national legislation and the institutional requirements.

## Author contributions

All authors listed have made a substantial, direct, and intellectual contribution to the work, and approved it for publication.

## References

[B1] Abdul-Rashid S. H. Sakundarini N. Ghazilla R. A. R. Thurasamy R. (2017). The impact of sustainable manufacturing practices on sustainability performance: Empirical evidence from Malaysia. *Int. J. Oper. Prod. Manag.* 37 182–204. 10.1108/IJOPM-04-2015-0223

[B2] Abid M. (2017). Does economic, financial and institutional developments matter for environmental quality? A comparative analysis of EU and MEA countries. *J. Environ. Manag.* 188 183–194. 10.1016/j.jenvman.2016.12.007 27984791

[B3] Ananda A. R. W. Astuty P. Nugroho Y. C. (2018). Role of green supply chain management in embolden competitiveness and performance: Evidence from Indonesian organizations. *Int. J. Supp. Chain Manag.* 7 437–442.

[B4] Anthony B. J. (2016). Green information systems integration in information technology based organizations: An academic literature review. *J. Soft Comput. Decis. Supp. Syst.* 3 45–66.

[B5] Arshad Ali A. Mahmood A. Ikram A. Ahmad A. (2020). Configuring the drivers and carriers of process innovation in manufacturing organizations. *J. Open Innov.* 6:154. 10.3390/joitmc6040154

[B6] Awan U. Kraslawski A. Huiskonen J. (2017). Understanding the relationship between stakeholder pressure and sustainability performance in manufacturing firms in Pakistan. *Proc. Manuf.* 11 768–777. 10.1016/j.promfg.2017.07.178

[B7] Aziziankohan A. Jolai F. Khalilzadeh M. Soltani R. Tavakkoli-Moghaddam R. (2017). Green supply chain management using the queuing theory to handle congestion and reduce energy consumption and emissions from supply chain transportation fleet. *J. Indust. Eng. Manag.* 10 213–236. 10.3926/jiem.2170

[B8] Barney J. B. (2012). Purchasing, supply chain management and sustained competitive advantage: The relevance of resource-based theory. *J. Suppl. Chain Manag.* 48 3–6. 10.1111/j.1745-493X.2012.03265.x

[B9] Cankaya S. Y. Sezen B. (2018). Effects of green supply chain management practices on sustainability performance. *J. Manufact. Technol. Manag.* 30 98–121. 10.1108/JMTM-03-2018-0099

[B10] Centobelli P. Cerchione R. Ertz M. (2020). Managing supply chain resilience to pursue business and environmental strategies. *Bus. Strategy Environ.* 29 1215–1246. 10.1002/bse.2428 24830282

[B11] Centobelli P. Cerchione R. Oropallo E. El-Garaihy W. H. Farag T. Al Shehri K. H. (2022). Towards a sustainable development assessment framework to bridge supply chain practices and technologies. *Sustain. Dev.* 30 647–663. 10.1002/sd.2262

[B12] Cerchione R. Bansal H. (2020). Measuring the impact of sustainability policy and practices in tourism and hospitality industry. *Bus. Strategy Environ.* 29 1109–1126. 10.1002/bse.2420

[B13] Chavez R. Yu W. Feng M. Wiengarten F. (2016). The effect of customer-centric green supply chain management on operational performance and customer satisfaction. *Bus. Strategy Environ.* 25 205–220. 10.1002/bse.1868

[B14] Chen Y.-S. (2008). The positive effect of green intellectual capital on competitive advantages of firms. *J. Bus. Ethics* 77 271–286. 10.1007/s10551-006-9349-1

[B15] Cherian J. Jacob J. (2012). Green marketing: A study of consumers’ attitude towards environment friendly products. *Asian Soc. Sci.* 8 117–126. 10.5539/ass.v8n12p117

[B16] Chin T. A. Tat H. H. Sulaiman Z. (2015). Green supply chain management, environmental collaboration and sustainability performance. *Procedia Cirp* 26 695–699. 10.1016/j.procir.2014.07.035

[B17] Choi D. Hwang T. (2015). The impact of green supply chain management practices on firm performance: The role of collaborative capability. *Operat. Manag. Res.* 8 69–83. 10.1007/s12063-015-0100-x

[B18] Dao V. Langella I. Carbo J. (2011). From green to sustainability: Information technology and an integrated sustainability framework. *J. Strateg. Inform. Syst.* 20 63–79. 10.1016/j.jsis.2011.01.002

[B19] David R. Muthini J. (2019). Influence of green supply chain management practices on procurement performance of private health institutions in Kenya: A case of aga khan hospital kisumu. *Strateg. J. Bus. Change Manag.* 6 1378–1396.

[B20] De D. Chowdhury S. Dey P. K. Ghosh S. K. (2020). Impact of lean and sustainability oriented innovation on sustainability performance of small and medium sized enterprises: A data envelopment analysis-based framework. *Int. J. Prod. Econ.* 219 416–430. 10.1016/j.ijpe.2018.07.003

[B21] Dedrick J. (2010). Green IS: Concepts and issues for information systems research. *Commun. Assoc. Inform. Syst.* 27:11. 10.17705/1CAIS.02711

[B22] Dhull S. Narwal M. (2016). Drivers and barriers in green supply chain management adaptation: A state-of-art review. *Uncertain Supply Chain Manag.* 4 61–76. 10.5267/j.uscm.2015.7.003

[B23] DiMaggio P. J. Powell W. W. (1983). The iron cage revisited: Institutional isomorphism and collective rationality in organizational fields. *Am. Sociol. Rev.* 40 147–160. 10.2307/2095101

[B24] Dubey R. Gunasekaran A. Ali S. S. (2015). Exploring the relationship between leadership, operational practices, institutional pressures and environmental performance: A framework for green supply chain. *Int. J. Prod. Econ.* 160 120–132. 10.1016/j.ijpe.2014.10.001

[B25] Fernando Y. (2017). An empirical analysis of eco-design of electronic products on operational performance: Does environmental performance play role as a mediator? *Int. J. Bus. Innovat. Res.* 14 188–205. 10.1504/IJBIR.2017.086285 35009967

[B26] Foo P.-Y. Lee V.-H. Tan G. W.-H. Ooi K.-B. (2018). A gateway to realising sustainability performance via green supply chain management practices: A PLS–ANN approach. *Expert Syst. Appl.* 107 1–14. 10.1016/j.eswa.2018.04.013

[B27] Gong R. Xue J. Zhao L. Zolotova O. Ji X. Xu Y. (2019). A bibliometric analysis of green supply chain management based on the Web of Science (WOS) platform. *Sustainability* 11 3459. 10.3390/su11123459

[B28] Govindan K. Diabat A. Shankar K. M. (2015). Analyzing the drivers of green manufacturing with fuzzy approach. *J. Clean. Prod.* 96 182–193. 10.1016/j.jclepro.2014.02.054 34746984

[B29] Gupta K. (2019). *Innovations In Manufacturing For Sustainability.* Berlin: Springer. 10.1007/978-3-030-03276-0

[B30] Habib M. A. Bao Y. Nabi N. Dulal M. Asha A. A. (2021). Impact of strategic orientations on the implementation of green supply chain management practices and sustainable firm performance. *MDPI* 13:340. 10.3390/su13010340

[B31] Hirsch P. M. (2008). “Been there, done that, moving on: Reflections on institutional theory’s continuing evolution,” in *Handbook of Organizational Institutionalism*, eds Greenwood R. Oliver C. Suddaby R. Andersson E. (Thousand Oaks, CA: Sage), 783–789. 10.4135/9781849200387.n34

[B32] Hitt M. A. Xu K. Carnes C. M. (2016). Resource based theory in operations management research. *J. Operat. Manag.* 41 77–94. 10.1016/j.jom.2015.11.002

[B33] Ikram A. Su Q. Fiaz M. Rehman R. U. (2018). Cluster strategy and supply chain management: The road to competitiveness for emerging economies. *Benchmarking* 25 1302–1318. 10.1108/BIJ-06-2015-0059

[B34] Jenkin T. A. Webster J. McShane L. (2011). An agenda for ‘Green’information technology and systems research. *Inform. Organ.* 21 17–40. 10.1016/j.infoandorg.2010.09.003

[B35] Kaur K. (2021). The effect of green product design and institutional pressures on manufacturing firms performances in malaysia : Implementation of reverse logistics products. *Int. J. Supply Chain Manag.* 6 12–30. 10.47604/ijscm.1292

[B36] Keles I. Bekimbetova T. (2013). Measuring attitudes towards ‘Green’purchases: A study of university students in Kyrgyzstan. *Univ. J. Indust. Bus. Manag.* 1 46–49. 10.13189/ujibm.2013.010204

[B37] Khan S. A. R. Jian C. Yu Z. Golpîra H. Kumar A. (2019). “Impact of green practices on Pakistani manufacturing firm performance: A path analysis using structural equation modeling Computational intelligence and sustainable systems,” in *Computational Intelligence and Sustainable Systems*, (Springer), 87–97. 10.1007/978-3-030-02674-5_6

[B38] Khan S. A. R. Qianli D. (2017). Impact of green supply chain management practices on firms’ performance: An empirical study from the perspective of Pakistan. *Environ. Sci. Pollut. Res.* 24 16829–16844. 10.1007/s11356-017-9172-5 28573559

[B39] Khoiruman M. Haryanto A. T. (2017). Green purchasing behavior analysis of government policy about paid plastic bags. *Indonesian J. Sustain. Account. Manag.* 1 31–39. 10.28992/ijsam.v1i1.25

[B40] Krejcie R. V. Morgan D. W. (1970). Determining sample size for research activities. *Educ. Psychol. Measur.* 30 607–610. 10.1177/001316447003000308

[B41] Kuenzi M. Brown M. E. Mayer D. M. Priesemuth M. (2019). Supervisor-subordinate (dis) agreement on ethical leadership: An investigation of its antecedents and relationship to organizational deviance. *Bus. Ethics Quart.* 29 25–53. 10.1017/beq.2018.14

[B42] Kuo T. C. Tseng M.-L. Lin C. H. Wang R.-W. Lee C.-H. (2018). Identifying sustainable behavior of energy consumers as a driver of design solutions: The missing link in eco-design. *J. Clean. Prod.* 192 486–495. 10.1016/j.jclepro.2018.04.250

[B43] Liobikienė G. Mandravickaitė J. Bernatonienė J. (2016). Theory of planned behavior approach to understand the green purchasing behavior in the EU: A cross-cultural study. *Ecol. Econ.* 125 38–46. 10.1016/j.ecolecon.2016.02.008

[B44] Muhammad F. Ikram A. Jafri S. K. Naveed K. (2020). Product innovations through ambidextrous organizational culture with mediating effect of contextual ambidexterity: An empirical study of IT and telecom firms. *J. Open Innovat.* 7:9. 10.3390/joitmc7010009

[B45] Neramballi A. Sequeira M. Rydell M. Vestin A. Ibarra M. (2017). “A comprehensive literature review of green supply chain management,” *Paper presented at the 2nd World Congress on Civil, Structural, and Environmental Engineering (CSEE Congress 2017), Barcelona, Spain, April 2-4, 2017.* (Barcelona) 10.11159/icesdp17.176

[B46] Oliver C. (1997). Sustainable competitive advantage: Combining institutional and resource-based views. *Strateg. Manag. J.* 18 697–713. 10.1002/(SICI)1097-0266(199710)18:9<697::AID-SMJ909>3.0.CO;2-C

[B47] Peattie K. (2001). Golden goose or wild goose? The hunt for the green consumer. *Bus. Strategy Environ.* 10 187–199. 10.1002/bse.292

[B48] Pigosso D. C. A. McAloone T. C. Rozenfeld H. (2014). “Systematization of best practices for ecodesign implementation,” *Paper presented at the DS 77: Proceedings of the DESIGN 2014 13th International Design Conference.* (Glasgow)

[B49] Rao P. Holt D. (2005). Do green supply chains lead to competitiveness and economic performance? *Int. J. Operat. Prod. Manag.* 25 898–916. 10.1108/01443570510613956

[B50] Recker J. (2016). “Toward a design theory for green information systems,” *Paper presented at the 2016 49th Hawaii International Conference on System Sciences (HICSS).* Koloa, HI: IEEE. 10.1109/HICSS.2016.556

[B51] Rodrigues V. P. Pigosso D. C. A. McAloone T. C. (2016). Process-related key performance indicators for measuring sustainability performance of ecodesign implementation into product development. *J. Clean. Prod.* 139 416–428. 10.1016/j.jclepro.2016.08.046

[B52] Sarkis J. Zhu Q. Lai K.-H. (2011). An organizational theoretic review of green supply chain management literature. *Int. J. Prod. Econ.* 130 1–15. 10.1016/j.ijpe.2010.11.010

[B53] Schaltegger S. Wagner M. (eds) (2017). “Managing and measuring the business case for sustainability: Capturing the relationship between sustainability performance, business competitiveness and economic performance,” in *Managing the Business Case for Sustainability: The Integration of Social, Environmental and Economic Performance* (London: Routledge), 1–27. 10.4324/9781351280525-1

[B54] Schöggl J.-P. Baumgartner R. J. Hofer D. (2017). Improving sustainability performance in early phases of product design: A checklist for sustainable product development tested in the automotive industry. *J. Clean. Prod.* 140 1602–1617. 10.1016/j.jclepro.2016.09.195

[B55] Seethamraju R. C. Frost G. (2019). “Deployment of Information Systems for Sustainability Reporting and Performance,”. *25th Americas Conference on Information Systems, AMCIS 2019, Cancún, Mexico, August 15-17, 2019* (Atlanta, GA)

[B56] Seles B. M. R. P. de Sousa Jabbour A. B. L. Jabbour C. J. C. Dangelico R. M. (2016). The green bullwhip effect, the diffusion of green supply chain practices, and institutional pressures: Evidence from the automotive sector. *Int. J. Prod. Econ.* 182 342–355. 10.1016/j.ijpe.2016.08.033

[B57] Shevchuk N. Oinas-Kukkonen H. (2016). “Exploring green information systems and technologies as persuasive systems: A systematic review of applications in published research,” In *CIS 2016 Proceedings.* (Dublin)

[B58] Tiwari S. Wee H.-M. Daryanto Y. (2018). Big data analytics in supply chain management between 2010 and 2016: Insights to industries. *Comput. Ind. Eng.* 115 319–330. 10.1016/j.cie.2017.11.017

[B59] Van Blerkom M. (2008). *Measurement and Statistics for Teachers.* Berlin: Routledge. 10.4324/9780203887868

[B60] Yang C.-S. Lu C.-S. Haider J. J. Marlow P. B. (2013). The effect of green supply chain management on green performance and firm competitiveness in the context of container shipping in Taiwan. *Transp. Res. Part E* 55 55–73. 10.1016/j.tre.2013.03.005

[B61] Zaid A. A. Jaaron A. A. Bon A. T. (2018). The impact of green human resource management and green supply chain management practices on sustainable performance: An empirical study. *J. Clean. Prod.* 204 965–979. 10.1016/j.jclepro.2018.09.062

